# Pneumonia severity, comorbidity and 1-year mortality in predominantly older adults with community-acquired pneumonia: a cohort study

**DOI:** 10.1186/s12879-014-0730-x

**Published:** 2015-01-08

**Authors:** Thomas Wesemann, Harald Nüllmann, Marc Andre Pflug, Hans Jürgen Heppner, Ludger Pientka, Ulrich Thiem

**Affiliations:** Department of Geriatrics, Marienhospital Herne, University of Bochum, Widumer Str. 8, D-44627 Herne, Germany; Department of Geriatrics, HELIOS Klinikum Schwelm, University of Witten/Herdecke, Schwelm, Germany; Department of Medical Informatics, Statistics and Epidemiology, University of Bochum, Bochum, Germany

## Abstract

**Background:**

In patients with community-acquired pneumonia (CAP), short-term mortality is largely dependent on pneumonia severity, whereas long-term mortality is considered to depend on comorbidity. However, evidence indicates that severity scores used to assist management decisions at disease onset may also be associated with long-term mortality. Therefore, the objective of the study was to investigate the performance of the pneumonia severity scores CURB-65 and CRB-65 compared to the Charlson Comorbidity Index (CCI) for predicting 1-year mortality in adults discharged from hospital after inpatient treatment for CAP.

**Methods:**

From a single centre, all cases of patients with CAP treated consecutively as inpatients between 2005 and 2009 and surviving at least 30 days after admission were analysed. The patients’ vital status was obtained from the relevant local register office. CURB-65, CRB-65 and CCI were compared using receiver operating characteristics (ROC) analysis.

**Results:**

Of 498 cases analysed, 106 (21.3%) patients died within 1 year. In univariate analysis, age ≥65 years, nursing home residency, hemiplegia, dementia and congestive heart failure were significantly associated with mortality. CURB-65, CRB-65 and CCI were also all associated with mortality at 1 year. ROC analysis yielded a weak, yet comparable test performance for CURB-65 (AUC and corresponding 95% confidence interval [CI] for risk categories: 0.652 [0.598-0.706]) and CCI (AUC [CI]: 0.631 [0.575-0.688]; for CRB-65 0.621 [0.565-0.677] and 0.590 [0.533-0.646]).

**Conclusions:**

Neither CURB-65 or CRB-65 nor CCI allow excellent discrimination in terms of predicting longer term mortality. However, CURB-65 is significantly associated with long-term mortality and performed equally to the CCI in this respect. This fact may help to identify CAP survivors at higher risk after discharge from hospital.

## Background

Community acquired pneumonia (CAP) is the major cause of mortality from infectious diseases in western countries. In 2011, around 20,000 patients in Germany died due to pneumonia and influenza [1]. It is notable that more than 18,000 of these patients were 65 or older at date of death [1]. The estimated national incidence of CAP ranges from 400,000 to 680,000 infections per year [2]. National and international guidelines recommend assessing the severity of CAP at initial presentation [3,4]. Several risk scores such as the CURB-65 [5] or the pneumonia severity index (PSI) [6] have been developed to assist initial management decisions. For CURB-65, for each risk factor present (confusion, urea >7 mmol/L, respiratory rate ≥30 per minute, low systolic [<90 mm Hg] or diastolic blood pressure [<60 mm Hg] and age ≥65 years), 1 point is awarded. An even simpler derivate, the CRB-65, which omits the urea criterion, is recommended and is in use. Both CURB-65 and CRB-65 have been validated for short-term mortality and permit a stratification of patients into 3 risk classes (‘low risk’, ‘intermediate risk’ and ‘high risk’) [7-11].

Besides remarkable hospital mortality, research from the recent years shows alarming long-term mortality among patients who were discharged as clinically recovered after a CAP episode [12-21]. Long-term mortality has proved to be strongly and independently associated with comorbidity, as studies consistently report [12,15,17-19]. A well-known method for classifying comorbid conditions is the Charlson Comorbidity Index (CCI) [22,23]. With respect to the number and severity of comorbid conditions, this method estimates the risk of dying in the following years. The CCI has been validated for short-term and long-term outcome in different study samples [24].

Apart from comorbidity, several studies report that pneumonia severity on admission has also an influence on long-term mortality [13,14,16,17,25]. These studies indicate that pneumonia severity scores may be helpful for the prediction of long-term outcome. Studies have focused mainly on the Pneumonia Severity Index (PSI) [13,14,16,17,25]. This rather complex index based on demographic characteristics, comorbid illness, physical examination and radiographic and laboratory findings is heavily weighted by age and comorbidity [6]. At present, it is not clear whether simpler pneumonia severity scores such as the CURB-65 or CRB-65 are also useful in the prediction of long-term outcome in CAP patients. Therefore, the aim of our study was to investigate the performance of the CURB-65 and CRB-65 pneumonia severity scores compared to the CCI for the prediction of 1-year mortality in adults discharged from hospital after inpatient treatment for CAP.

## Methods

### Setting and study population

We conducted a retrospective study at the Marienhospital Herne, Herne, Germany. Some study details and results on short-term mortality have already been published [26]. Data of all adult inpatients ≥ 18 years hospitalised between 2005 and 2009 with CAP were extracted from the hospital’s CAP database. This database was established in the year 2005 as part of the German national quality assurance programme and includes information about confusion due to pneumonia, respiratory rate and blood pressure upon hospital admission, age, nursing home residency, immobility, mechanical ventilation and whether or not treatment was discontinued or not performed. These variables are assessed and documented during the hospital stay of the given patient. Additional International Classification of Diseases (ICD-10) codes as well as laboratory values documented during the hospital stay were obtained from the hospital information technology system. On the basis of this data, CURB-65 and CRB-65 [5] were calculated, as previously described. The CCI is a weighted comorbidity index [22] that lists nineteen diseases and conditions with major impact on survival, for example heart, liver and kidney failure, chronic lung diseases, diabetes, hemiplegia, cancer, leukemia and AIDS. For each condition, between one and six points are awarded and summed up for the summary score. Higher score sums indicate higher mortality [22]. To retrieve the conditions from the administrative data set, we used the ICD-10 code list as published by Quan et al. [23]. Information about the patients’ vital status and/or date of death was obtained from the relevant local register office.

To validate CAP diagnosis, all cases in the database were reviewed. We included those cases with typical clinical symptoms of pneumonia (at least 1 of the symptoms dyspnoea, cough, new sputum production or temperature ≥ 38.0°C) and with either newly shown pulmonary infiltrate on chest x-ray or elevated biochemical markers of inflammation within 48 hours of hospital admission with no evidence of other infectious diseases. Patients who had undergone radiotherapy or chemotherapy within 28 days prior to admission or with known immunodeficiency of other causes were excluded. Patients with nosocomial pneumonia and pneumonia due to other causes, e.g. stenosis-induced pneumonia or pneumonia following pulmonary embolism, as well as patients suffering from acute exacerbation of chronic obstructive disease were also excluded. Patients who had not undergone or discontinued treatment, those referred from other hospitals and patients on mechanical ventilation upon admission were considered ineligible for inclusion. The study was restricted to inpatients who survived the initial admission for CAP for at least 30 days.

### Sample size and statistical analysis

For the purpose of this study, we used a dichotomous risk factor for 1-year mortality, ‘low risk’ versus ‘high risk’, as defined, for example, by CCI or CURB-65. On the basis of data from Hsu et al. [27], we assumed a mortality of 20% in the low-risk group and 32% in the high-risk group, corresponding to a relative risk of 1.6. Using Fisher’s exact test for independent samples with a significance level of 5% and a power of 80%, we calculated about 226 cases per group or about 452 patients in total.

Categorical variables are presented in absolute numbers and proportions, continuous variables with median, mean and range. Risk stratification for all patients was calculated on the basis of CURB-65, CRB-65 and CCI by score points and risk categories, respectively. The association between categorical variables was assessed using a chi-square test. For multivariate analysis, logistic regression analysis was used, with one model for each of the scores in use. In all models, death within 365 days after hospital admission was the dependent variable, coding ‘1’ for death and ‘0’ for being alive. As explanatory variables, we used age (dichotomous, using median age as cut-off), sex and the score in three categories, respectively. Receiver operating characteristics (ROC) curves were constructed for score points and risk categories of CURB-65, CRB-65 and CCI. The area under the ROC curve (AUROC) was calculated with corresponding 95% confidence intervals (CI). As the primary endpoint, we use death from any cause at 1 year after hospital admission for CAP.

To calculate the sample size, we used StatsDirect Software (version 2.7.9, 2012, StatsDirect Ltd., UK). All other analyses were performed with SPSS for Windows (version 21, 2012, IBM Inc., USA).

## Results

Data of 587 inpatients with CAP were extracted from the hospital´s CAP database. Patients referred from other hospitals (4 patients), those who either discontinued or did not undergo treatment (18 patients) and patients on mechanical ventilation on admission (six patients) were excluded. Of 559 remaining patients, 61 died within 30 days after admission, leaving 498 patients for analysis. Baseline characteristics of the study sample are presented in Table 1.

**Table 1:** **Characteristics of the study sample**

**Characteristics**	**Total (n = 498)**		**Survivors (n = 392)**		**Non-survivors (n = 106)**	
	**n**	**%**	**n**	**%**	**n**	**%**
Age ≥65 years^1^	379	76.1	279	71.2	100	94.3
Sex (male)	279	56.0	230	58.7	49	46.2
Nursing home residency^1^	111	22.3	71	18.1	40	37.7
Hemiplegia^1^	36	7.2	20	5.1	16	15.1
Congestive heart failure^1^	105	21.1	73	18.6	32	30.2
Chronic lung disease	146	29.3	123	31.4	23	21.7
Chronic kidney disease	128	25.7	99	25.3	29	27.4
Diabetes	144	28.9	106	27.0	38	35.8
Cerebrovascular disease	98	19.7	59	15.1	39	36.8
Dementia^1^	140	28.1	89	22.7	51	48.1

^1^Statistically significant difference between survivors and non-survivors (p < 0.05).

The patients’ median age was 77.7 years (mean 73.0, range 18.1 to 104.2). 279 patients were male (56.0%). Functional impairment was prevalent in the study sample, with 111 patients being nursing home residents (22.3%). Comorbid conditions such as congestive heart failure (105 patients, 21.1%), dementia (140 patients, 28.1%), and diabetes mellitus (144 patients, 28.9%) were also frequent. 417 patients (83.7%) scored at least 1 point according to the CCI and 303 individuals (60.8%) had advanced comorbidity. In terms of pneumonia severity, 205 patients (41.2%) were classified into the CURB-65-defined low-risk group, whereas 293 patients (58.8%) were at intermediate or high risk. With CRB-65, 86 patients scored 0 points and hence were classified into the low-risk group.

Of 498 patients, 106 (21.3%) died within 1 year. Table 1 illustrates the association of patients’ characteristics and comorbid conditions among survivors and those who died within 1 year of admission. Univariate analysis showed a statistically significant association with 1-year CAP mortality for age ≥65 years, nursing home residency, hemiplegia, dementia and congestive heart failure. The association between CCI and the CURB-65 and CRB-65 pneumonia severity scores with 1-year mortality was also statistically significant. Overall, 1-year mortality increased with higher score points and risk categories for CCI as well as for CURB-65 and CRB-65 (Table 2). These findings were similar after adjustments for age and gender (data not shown).

**Table 2:** **1-year mortality stratified by score points and risk categories**

**Severity score**	**Score points**	**Total**	**Mortality**	
		**(n = 498)**	**n**	**%**
CURB-65	0	72	3	4.2
	1	133	14	10.5
	2	177	56	31.6
	3	97	28	28.9
	4	18	5	27.4
	5	1	0	0.0
CRB-65	0	86	4	4.7
	1	248	54	21.8
	2	131	39	29.8
	3	30	8	26.7
	4	3	1	33.3
CCI	0	81	6	7.4
	1	114	14	12.3
	2	94	24	25.5
	3	81	25	30.9
	4	52	11	21.2
	5	43	17	39.5
	6	17	2	11.8
	7	8	3	37.5
	8	5	1	20.0
	9	3	3	100.0
**Severity score**	**Score points**	**Total**	**Mortality**	
		**(n = 498)**	**n**	**%**
CURB-65	Low risk	205	17	8.3
	Intermediate risk	177	56	31.6
	High risk	116	33	28.4
CRB-65	Low risk	86	4	4.7
	Intermediate risk	379	93	24.5
	High risk	33	9	27.3
CCI	Low risk	195	20	10.3
	Intermediate risk	175	49	28.0
	High risk	128	37	28.9

Definition of risk categories:

CURB-65: low risk = 0-1 points, intermediate risk = 2 points, high risk = 3-5 points.

CRB-65: low risk = 0 points, intermediate risk = 1-2points, high risk = 3-4 points.

CCI: low risk = 0-1 points, intermediate risk = 2-3 points, high risk ≥ 3points.

Chi-square test for all associations: p < 0.001.

**Figure 1:**
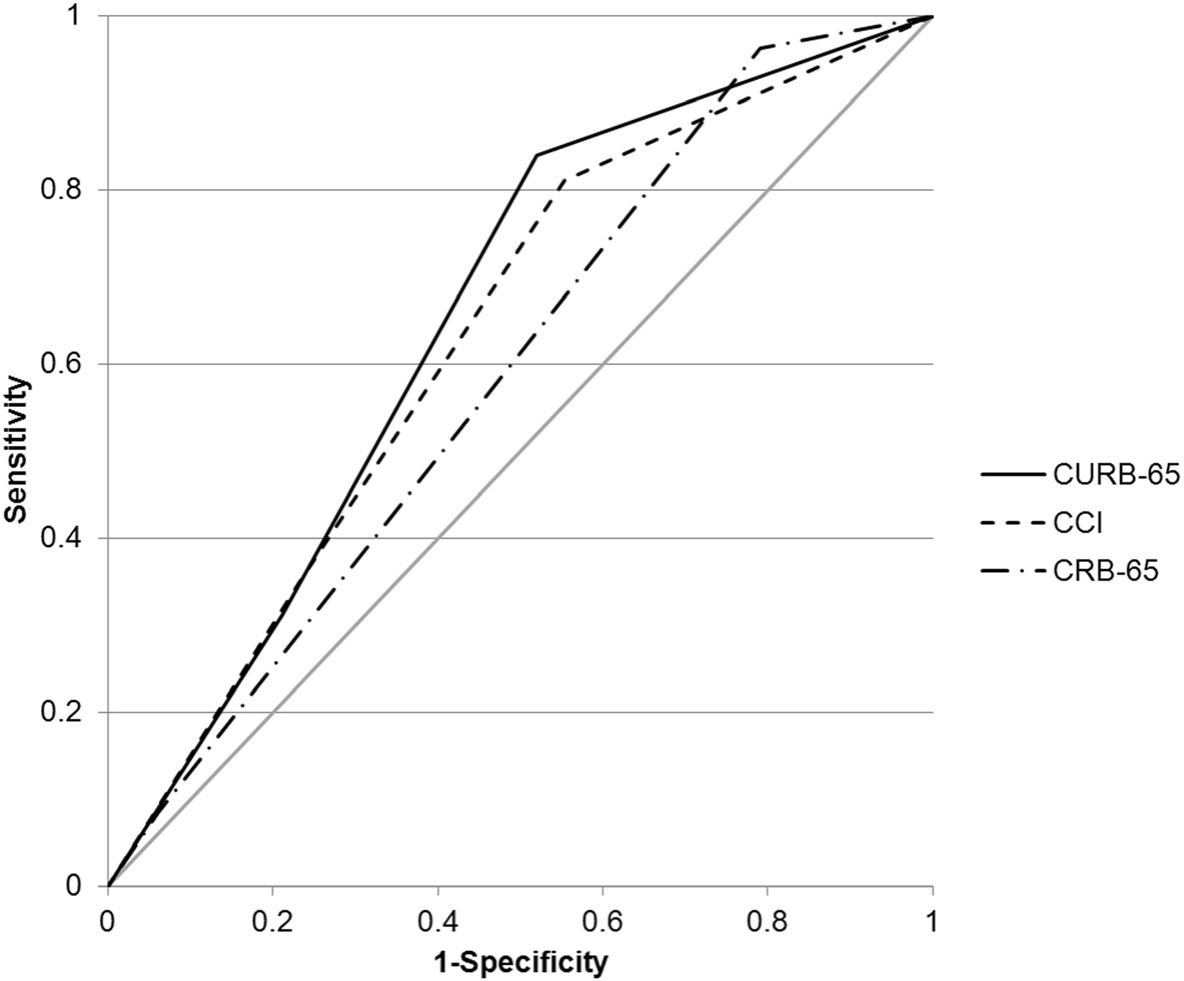
**Receiver operating characteristics (ROC) curves for risk categories.**

ROC analysis yielded a weak, yet comparable test performance for CURB-65 and CCI. AUROC values and corresponding CI for score points and risk categories, respectively, were as follows: for CURB-65 0.658 [0.606-0.711] and 0.652 [0.598-0.706]; for CRB-65 0.621 [0.565-0.677] and 0.590 [0.533-0.646]; and for CCI 0.647 [0.592-0.702] and 0.631 [0.575-0.688]. The ROC curves for risk categories are shown in Figure 1.

## Discussion

The main finding of our study is that none of the scores performed satisfactorily in predicting 1-year mortality after inpatient treatment of predominantly older adults with CAP. It is noteworthy, however, that the simple CURB-65 pneumonia severity score appears to be on par with the CCI in this respect. This result may be surprising, given the importance of comorbidity for the long-term outcome of CAP [12,15,17-19]. Our analysis confirms the role of comorbid conditions as assessed by the CCI, as already highlighted by previous studies. In a recent analysis of 1,117 patients, Capelastegui et al. showed that the CCI is an independent predictor of 90-day mortality in CAP [28]. Bordon et al. used a modified, differently weighted form of the CCI that was able to predict long-term outcome in a CAP study sample with an average follow-up of 7.5 years [15].

Contrary to the CCI, the CURB-65 and CRB-65 scores are viewed as markers of disease severity on admission similar to the PSI. Each of the pneumonia severity scores is well validated for predicting 30-day CAP mortality [7]. The simpler CURB-65 and CRB-65 scores differ from the more complex PSI, as they do not heavily weight age nor include comorbidity. Mortensen et al. demonstrated that a higher initial PSI class is significantly associated with decreased long-term survival [18]. Similarly, in a recent study with 3,284 CAP patients, Johnstone et al. showed that long-term outcome is strongly associated with the initially calculated PSI class [16]. They hypothesised that the PSI may be a marker of an individual’s overall health status and risk of death from an episode of acute illness rather than a true marker of pneumonia severity [16]. Another explanation could be that pneumonia severity is dependent on the health status before pneumonia onset. This would explain our finding that scores such as CURB-65 and CRB-65, although they do not include variables of comorbidity, are also related to 1-year mortality after CAP. Our finding is in line with one study reporting an independent association of the CURB score, a variant omitting the age criterion, with adverse outcomes 90 days after hospitalisation for CAP [28], and a further study by Krüger et al. showing an association between 180-day mortality and CRB-65 [29].

Although not satisfying as prognostic tests, the relationship between CURB-65 and CRB-65 and 1-year mortality may be helpful for clinicians, as no other gold standard is available. Compared to more complex tools such as CCI or PSI, the advantages of the CURB-65 and CRB-65 are striking: the scores are easy to calculate on admission even in a busy emergency department; CURB-65 and CRB-65 scores are already implemented in current guidelines for site-of-care decisions [3,4]; for most inpatients, the calculation is performed on admission, so that the calculated score is readily available on hospital discharge.

The fact that surveillance needs to be improved has once again been demonstrated with our study. We found that more than one-fifth of patients who survived the index hospitalisation due to CAP died within 1 year after admission. Hsu et al. found that 17% of CAP hospital survivors and 34.4% of hospital survivors of health care-associated pneumonia (HCAP) died within the first year following discharge [27]. Kaplan et al. reported a 1-year mortality of 33.6% among 141,498 patients aged 65 years or older who survived hospital treatment for pneumonia [19]. In conclusion, long-term-mortality of inpatients surviving hospitalisation for CAP is striking and found to be sustainably higher than that of the general population [12,20].

Apart from CCI, CURB-65 and CRB-65, several other variables were associated with increased 1-year mortality in our sample, including age ≥65 years, nursing home residency, hemiplegia, dementia and congestive heart failure. Nursing home residency and impaired cognitive function have already been described as risk factors for unfavourable long-term outcome in CAP patients, along with congestive heart failure and cardiovascular disease [12,17,18,27,30,31]. In a recent study with 897 patients, Yende et al. suggested an explanation for the link between pneumonia and cardiovascular death [14]. They found that elevated haemostasis markers are common in CAP patients on hospital discharge. This postinflammatory prothrombotic state was offered as an explanation for the increased risk of all causes of mortality and cardiovascular death over 1 year after hospitalisation for CAP. If so, surveillance should focus on patients of advanced age and with functional deficits as well as those with cardiovascular comorbidity.

Several limitations of the current study should be acknowledged. First, we performed a retrospective study, and we used registry data for analysis. However, we do not believe that this affects the validity of our findings. The data in use were mainly assessed and documented during the hospital stay of a given patient. All variables of the pneumonia severity scores were included, with the exception of blood urea levels. As blood urea is part of the standard laboratory measurements obtained for any hospital admission, urea values could be retrieved from the hospital’s information technology system for all cases. The CCI was calculated using ICD-10 codes from administrative data with a well-validated method. Finally, the primary endpoint, death from any cause 1 year after admission, was assessed using data from the city’s local register office, which is reliable even when performed retrospectively.

Second, only inpatients with pneumonia were included in our analysis. Current guidelines recommend outpatient treatment for CAP only for CURB-65 or CRB-65-defined low-risk patients and in the absence of advanced comorbidity. It is therefore unlikely that patients from risk groups other than the low-risk group were missed. However, this cannot be verified with our data, and hence our findings cannot be transferred to outpatients with CAP without reservation.

Third, findings were made in predominantly elderly patients with high prevalence of comorbid diseases and from a single centre only. Therefore, our findings also need to be validated in other samples and settings.

## Conclusions

In conclusion, our findings demonstrate substantial 1-year mortality in hospital survivors of CAP. Neither CURB-65 or CRB-65 nor CCI allow excellent discrimination in terms of predicting longer term mortality. Despite its simplicity, CURB-65 is significantly associated with long-term mortality and performed equally to the CCI in this respect. This fact may help to identify patients at higher risk after discharge from hospital. Further research is needed on prognostic markers as well as surveillance and rehabilitation for CAP patients who are considered clinically cured to reduce long-term mortality.

## Consent

Written informed consent was not obtained, because the hospital’s CAP database only contained anonymous data that were used in retrospect. The information on vital status received from the local register office was added by means of another anonymised file using a study specific patient identifier. Detailing this procedure, we applied for ethics approval. The ethics committee of the University of Bochum approved the study without any objection (registration no. 4376-12).
